# The Novel Aminomethylcycline Omadacycline Has High Specificity for the Primary Tetracycline-Binding Site on the Bacterial Ribosome

**DOI:** 10.3390/antibiotics5040032

**Published:** 2016-09-22

**Authors:** Corina G. Heidrich, Sanya Mitova, Andreas Schedlbauer, Sean R. Connell, Paola Fucini, Judith N. Steenbergen, Christian Berens

**Affiliations:** 1 Microbiology, Department of Biology, Friedrich-Alexander-Universität Erlangen-Nürnberg, 91058 Erlangen, Germany; corina.heidrich@gmail.com (C.G.H.); sanya.mitova@hotmail.de (S.M.); 2 Structural Biology Unit, CIC bioGUNE, 48160 Derio, Bizkaia, Spain; aschedlbauer@cicbiogune.es (A.S.); sean.connell@gmail.com (S.R.C.); pfucini@gmail.com (P.F.); 3 IKERBASQUE, Basque Foundation for Science, 48013 Bilbao, Spain; 4 Paratek Pharmaceuticals Inc., King of Prussia, PA 19406, USA; judith.steenbergen@paratekpharma.com; 5 Institute of Molecular Pathogenesis, Friedrich-Loeffler-Institut, 07743 Jena, Germany

**Keywords:** tetracycline, tigecycline, omadacycline, tetracycline resistance, antibiotics, antibiotic resistance, chemical probing, ribosome structure

## Abstract

Omadacycline is an aminomethylcycline antibiotic with potent activity against many Gram-positive and Gram-negative pathogens, including strains carrying the major efflux and ribosome protection resistance determinants. This makes it a promising candidate for therapy of severe infectious diseases. Omadacycline inhibits bacterial protein biosynthesis and competes with tetracycline for binding to the ribosome. Its interactions with the 70S ribosome were, therefore, analyzed in great detail and compared with tigecycline and tetracycline. All three antibiotics are inhibited by mutations in the 16S rRNA that mediate resistance to tetracycline in *Brachyspira hyodysenteriae*, *Helicobacter pylori*, *Mycoplasma hominis,* and *Propionibacterium acnes*. Chemical probing with dimethyl sulfate and Fenton cleavage with iron(II)-complexes of the tetracycline derivatives revealed that each antibiotic interacts in an idiosyncratic manner with the ribosome. X-ray crystallography had previously revealed one primary binding site for tetracycline on the ribosome and up to five secondary sites. All tetracyclines analyzed here interact with the primary site and tetracycline also with two secondary sites. In addition, each derivative displays a unique set of non-specific interactions with the 16S rRNA.

## 1. Introduction

Typical tetracyclines [1], like tetracycline (TET) or tigecycline (TGC), inhibit bacterial protein biosynthesis by binding to the 30S ribosomal subunit [2,3,4,5] and preventing stable accommodation of the EF-Tu-GTP-aa-tRNA complex at the ribosomal A-site [4,6]. Tetracyclines have broad-spectrum activity against many infectious disease agents, including Gram-negative and Gram-positive bacteria, intracellular pathogens, and even protozoan parasites (summarized in reference [7]). This, their low cost of production [8], and the absence of major adverse side-effects have led to their widespread application—not only for treating human and animal infections, but also as prophylactic or growth-promoting agents in animal feed [9]. Unfortunately, the extensive use of tetracyclines has severely limited their efficacy as antibiotics due to the concomitant emergence and spread of microbial resistance. Roughly 50 different determinants currently mediate resistance to the older clinically established tetracyclines. They have been grouped into two major mechanisms, drug efflux and ribosome protection, and two minor mechanisms, modification of the ribosomal target (16S rRNA mutations) and enzymatic inactivation [8,9,10].

**Figure 1:**
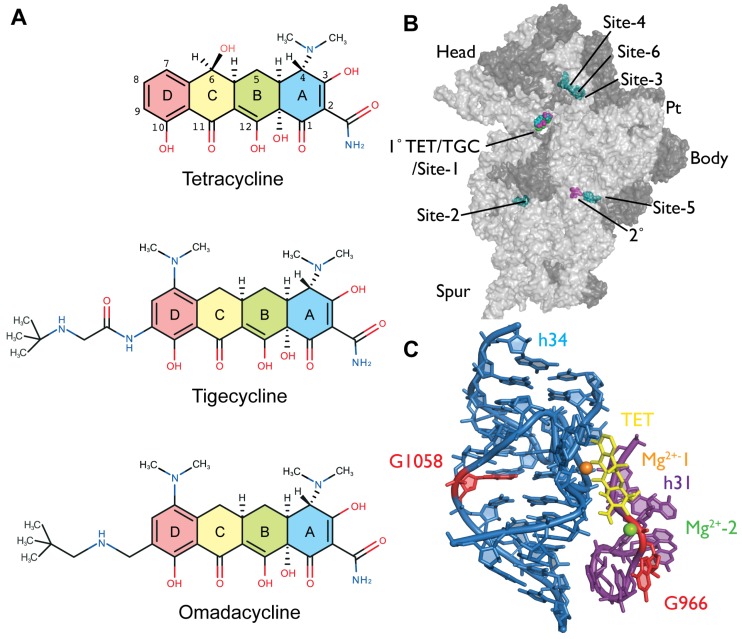
Tetracycline, tigecycline and omadacycline, the tetracycline binding sites and proximity of tetracycline rRNA resistance mutations to the primary binding site. (**A**) The chemical structures of tetracycline, tigecycline, and omadacycline drawn schematically with their common backbone ring structures (rings A–D) colored distinctly. Carbon atom assignments for the 4-ring backbone are indicated on tetracycline; (**B**) The primary and secondary tetracycline binding sites as observed in X-ray crystallography studies [2,3,4] are shown on the structure of the 30S ribosomal subunit (rRNA, light grey surface; ribosomal-proteins, dark grey surface). The primary (1°) and secondary (2°) TET binding sites observed by Brodersen et al. are colored pink, the primary (1°) site described by Jenner et al. is green but largely obscured underneath TET, and the TET binding sites 1–6 observed by Pioletti et al. are colored blue and labeled distinctly. The head, spur, platform (Pt) and body 30S subunit landmarks are labeled. (**C**) The primary tetracycline binding site according to Jenner et al. [4] is illustrated showing the two rRNA bases, G1058 and G966, whose mutation results in tetracycline resistance.

The increasing prevalence of tetracycline-resistant bacteria has triggered the development of new tetracycline derivatives, which are modified at positions C-7 and C-9 of the tetracycline D-ring (Figure 1A) and are highly active against organisms carrying the major resistance determinants.

The first representative of this third generation to have been approved by the FDA is tigecycline, a glycylcycline (Figure 1A) [11]. Another representative in clinical development is the semi-synthetic 9-aminomethylcycline omadacycline (Figure 1A) [12]. Both display potent activity against Gram-positive and Gram-negative bacteria, including strains carrying efflux and ribosome protection resistance determinants [13,14]; however, it has been shown that TGC remains susceptible to the minor tetracycline resistance mechanisms [15,16]. These new tetracycline derivatives inhibit bacterial protein biosynthesis and compete with TET for binding to the ribosome [4,13,17]. The binding of TET and TGC to the ribosome has been characterized using X-ray crystallography [2,3,4,5] showing that both bind to overlapping sites in the ribosomal A-site (primary (1°)/site-1; Figure 1B) [4]. Interestingly TET, but not TGC, was shown in two studies [2,3] to bind to several secondary sites (labeled secondary (2°) and sites 2–6; Figure 1B) that are consistent with previous biochemical investigations (reviewed in [18]).

The structural basis for the interaction of omadacycline (OMC) with the ribosome is uncharacterized. OMC is currently in clinical development for treatment of acute bacterial skin and skin structure infections and community-acquired bacterial pneumonia. It also compared favorably with linezolid in a randomized, investigator-blind, multicenter phase 2 trial for complicated skin and skin structure infections [19]. Due to its activity profile, the oral availability, and because OMC appears to be well-tolerated by patients, this aminomethylcycline has the potential to become an effective agent for treatment of serious infections. A thorough understanding of how OMC acts mechanistically will help to better evaluate its strengths as a therapeutic agent, as well as its limitations. OMC competes with TET for binding to the ribosome [4,13], but it is not clear if this competition occurs at the primary site [2,3,4] or at one or more of the secondary sites [2,3] (Figure 1B). Genetic analysis with 16S rRNA tetracycline-resistance mutations [20,21,22,23,24] and chemical probing [15,25] can identify and compare the TET, TGC, and OMC binding sites on the 16S rRNA. We, therefore, used these methods for the identification and characterization of OMC binding sites on *Escherichia coli* 70S ribosomes and compared them with binding sites for TET and TGC.

## 2. Results

### 2.1. Omadacycline Is Susceptible to 16S rRNA Mutations Conferring TET Resistance

Although it is known that OMC competes with TET for binding to the ribosome [13], it is unclear if this competition occurs at the primary and/or the many secondary tetracycline binding sites [3] (Figure 1B). To genetically address the specificity of the ribosome-OMC interaction, we determined the susceptibility of strains harboring tetracycline-resistance mutations that surround the primary tetracycline-binding site (Figure 1C). These 16S rRNA mutations include (1) the 1058 G→C (helix 34, h34) exchange, found in *Propionibacterium acnes* and *Brachyspira hyodysenteriae* [20,22], and (2) the 966 G→U (helix 31, h31) transversion identified in *Helicobacter pylori* [21] (Figure 1C). These mutations were introduced into *E. coli* TA527 [26], a strain that lacks all seven chromosomal rRNA operons, but instead carries a single plasmid-borne rRNA operon [27]; this allows 16S rRNA resistance mutations to be studied without any interfering wild type background. As summarized in Table 1, we determined the minimal inhibitory concentrations (MICs) of the *E. coli* quality control strain ATCC-25922, as well as *E. coli* TA527, carrying either wild-type (pKK3535) or mutant (pKK966U and pKK1058C) 16S rRNA genes, for the antibiotics TET, TGC, and OMC.

The MIC values were determined by the agar dilution method according to CLSI standards, except that the plates were incubated for 40 h. The permitted MIC range of the quality control strain ATCC-25922 is 0.5–2 µg/mL for tetracycline and 0.03–0.25 for tigecycline. Fold-increases in MIC values in strains with tetracycline-resistant ribosomes are given in parentheses; nd: not determined.

When compared to *E. coli* TA527 carrying a wild-type rRNA operon, both mutants showed a 4- to 8-fold increase in their MIC, indicating that all three drugs (TET, TGC, and OMC) are susceptible to 16S rRNA resistance mutations. As these mutations cluster around the primary tetracycline-binding site (Figure 1C), it is highly likely that OMC binds to this site similar to TET and TGC.

### 2.2. Chemical Probing Indicates That OMC Binds Specifically to the Primary TET Binding Site

To further establish that OMC binds to the bacterial ribosome at a site corresponding to the primary tetracycline binding site, we employed chemical probing (dimethyl sulfate (**DMS**) and Fe^2+^-mediated Fenton cleavage) to map the interaction of OMC on the 16S rRNA. In the first set of experiments, DMS modification of the 16S rRNA was carried out in the presence of TET, TGC, and OMC to test if the three drugs show overlapping modification patterns. Empty 70S ribosomes (0.5–0.6 µM) from *E. coli* CAN/20-E12 were treated with DMS in the presence of TET, TGC or OMC at concentrations ranging from 0.3 to 300 µM.

**Figure 2:**
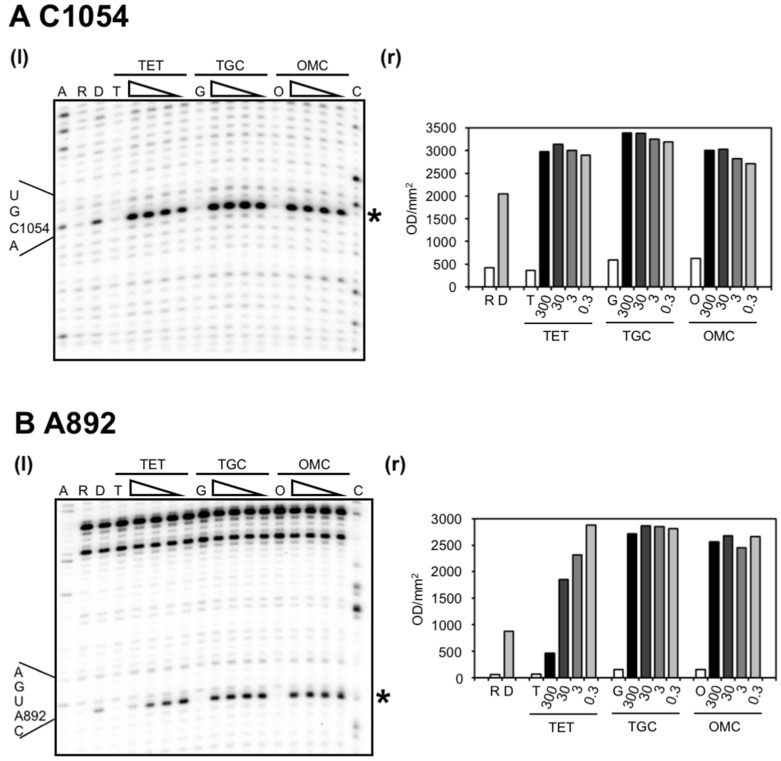
TET, TGC, and OMC affect DMS modification of bases in the16S rRNA. Empty *E. coli* 70S ribosomes (0.5–0.6 µM) were incubated with varying amounts of TET, TGC or OMC and methylated with DMS. Modification of nucleotides (**A**) C1054 and (**B**) A892 was detected by primer extension and analyzed by electrophoresis on denaturing 6% polyacrylamide gels, sections of which are shown in the panels (l) left of the plots (r) showing their respective quantification. The dideoxy sequencing lanes are indicated with A and C; the unmodified RNA with R; the unmodified rRNA in the presence of the antibiotics TET, TGC or OMC with T, G, and O respectively; the DMS-modified RNA in the absence of antibiotics with D; and the DMS modified RNA in the presence of antibiotic is indicated with wedges under the TET, TGC, OMC headers where the wedge represents the presence of antibiotics at 300, 30, 3, and 0.3 µM. The extent of DMS modification of the rRNA in the presence of increasing amounts of antibiotic was quantitated in a phosphorimager and is shown below the gel sections with a comparison to the control DMS-modified RNA in the absence of antibiotics (lanes designated as “D”). Quantification was adjusted for loading differences by normalization with regions unaffected by TET, TGC or OMC.

The C1054 (h34) enhancement (~1.5-fold), characteristic of tetracycline binding to the primary binding site, was observed for all compounds—even at the lowest concentration tested (0.3 µM; Figure 2A). In contrast, the protection of A892 from DMS methylation, which is indicative of binding to the secondary site near h27 of the 16S rRNA, was only detected with TET, and not with TGC or OMC (Figure 2B). Specifically, quantification showed that the intensity of the signal corresponding to A892 steadily decreased at TET concentrations from 0.3 µM up to 300 µM yielding a 6-fold reduction, while over a similar 1000-fold difference in concentration, TGC and OMC did not affect signal intensity (Figure 2B). We also observed a signal enhancement in the presence of the antibiotics. This was not seen with the previous protocol [15]. Since TET displayed a concentration-dependent decrease in signal intensity at A892, this initial enhancement might be caused indirectly by a structural change due to antibiotic binding to another site which then affects A892 exposure to DMS.

**Figure 3:**
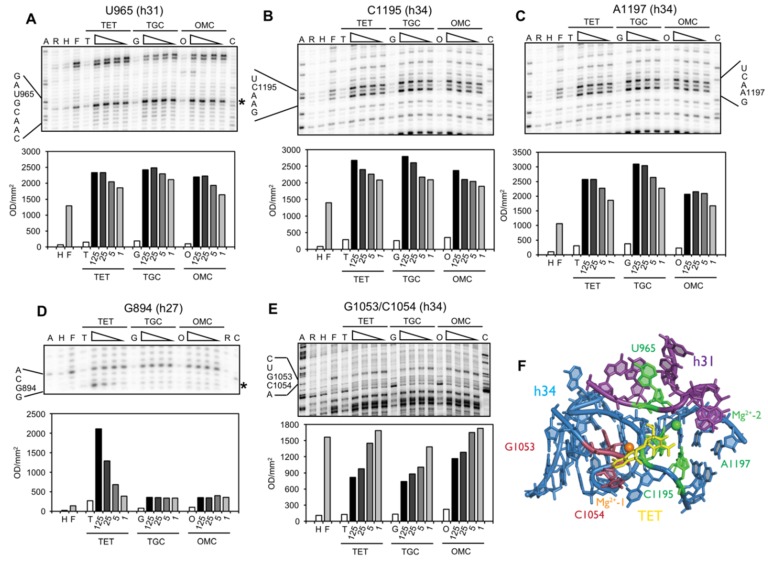
Fe^2+^-complexed with TET, TGC or OMC affects cleavage of bases in the 16S rRNA. Empty *E. coli* 70S ribosomes (2 µM) were incubated with increasing amounts of Fe^*2+*^-complexed TET, TGC or OMC (1–125 µM) and incubated with sodium ascorbate and hydrogen peroxide. Sites of cleavage were detected by primer extension and analyzed by electrophoresis on denaturing 6% polyacrylamide gels, sections of which are shown above the plots of their respective quantification. Dose-dependent changes in cleavage intensity were found at nucleotides (**A**) U965, (**B**) C1195, (**C**) A1197, (**D**) G894, and (**E**) G1053/C1054. The dideoxy sequencing lanes are indicated with A and C; the unmodified RNA with R; Fe^2+^ incubated rRNA in the absence of sodium ascorbate and hydrogen peroxide with H; Fenton-cleaved rRNA in the absence of antibiotics with F; unmodified rRNA in the presence of 125 µM antibiotic; TET, TGC, OMC with T, G, and O, respectively; Fenton-cleaved rRNA in the presence of the respective antibiotic under the TET, TGC, and OMC headers where the wedge represents the presence of 125, 25, 5, and 1 µM of the respective antibiotic. The extent of rRNA cleavage in the presence of increasing amounts of antibiotic was quantified in a phosphorimager and is shown below the gel sections with a comparison to the control Fenton-cleaved rRNA in the absence of antibiotic (shown in lanes designated “F”). Quantification was adjusted for loading differences by normalization to regions unaffected by TET, TGC or OMC. Note an identical gel slice is shown in panels B and C as the specified nucleotides are close in primary sequence. (**F**) Sites of increased (green: U965, C1195, and A1197) and decreased (red C1053, C1054) Fenton cleavage in the presence of the respective antibiotic within the primary tetracycline binding site [4] are shown. The 16S rRNA helices, h31 and h34, are colored purple and blue, respectively while the Mg^2+^ coordinated by tetracycline rings B and C is colored orange and the Mg^2+^ coordinated near tetracycline ring A is colored green. RNA residues are numbered according to the *E. coli* sequence.

In a second series of experiments, the interaction of OMC with the 16S rRNA was characterized and compared to that of TET and TGC using Fe^2+^-mediated Fenton cleavage. TET generally chelates a Mg^2+^ ion using the polar face of rings B and C and this Mg^2+^ ion is an important component of the TET binding pocket (Figure 1; Mg^2+^-1) [2,3,4,5]. In this approach the Mg^2+^ ion is substituted with an Fe^2+^ ion (Fe^2+^ has a 30–500-fold higher affinity to tetracycline than Mg^2+^ [28]) such that when the TET-Fe^2+^ chelate binds to the ribosome it can be used to generate Fe^2+^-dependent hydroxyl radicals (See Material and Methods) that cleave the rRNA in the local environment. Previously, this approach successfully mapped TET binding sites on the Tet repressor protein TetR [29] and the TET efflux protein TetA [30], as well as TET and TGC binding sites on the ribosome [15]. It is important to note that the Fe^2+^ and Mg^2+^ in the TET-chelate complex are considered isostructural [31] as Fe^2+^ can substitute for Mg^2+^ to induce the Tet repressor [29]. Using this approach, we probed empty ribosomes from *E. coli* CAN/20-E12 (2 µM) in the presence of TET, TGC, and OMC (1 to 125 µM) (Figure 3 and Figure S1). 

Similar to the DMS probing results, all three drugs showed overlapping cleavage patterns at the primary tetracycline binding site, whereas only TET mapped to the secondary binding site near h27 of the 16S RNA (Figure 3). Specific quantification of the cleavage sites seen in Figure 3 show that all three drugs enhanced the cleavage of U965 (h31), C1195 (h34), and A1197 (h34), while G1053 (h34) and C1054 (h34) were protected from cleavage. In contrast, only TET was observed to enhance cleavage of a residue close to a secondary binding site (G894, h27; Figure 3D). To validate that Fe^2+^ was interacting with a similar site as Mg^2+^, we performed a Mg^2+^ competition experiment and showed that Fe^2+^ dependent cleavage was reduced at all specific and non-specific cleavage sites identified for TET, TGC, and OMC, reaching intensities close to the background level (Figure S2). It is interesting to note that we also observed sixteen additional cleavage sites, but only at high antibiotic concentrations (≥25 µM; Figure S3). This is the first time that such frequent and idiosyncratic cleavage sites have been observed for tetracycline derivatives. They likely represent non-specific sites given that tetracyclines are known to bind non-specifically to RNA [32] particularly at concentrations above 40 µM [33]. Nevertheless, many map to sites (summarized in Table 2) that have been published either as tetracycline-affected (G242-G247, A279; G682/G683, U692/G693, A702/G703; G1166-A1169) or as interaction sites of molecules that are affected by tetracycline binding, like tRNA (U531/A532; G682/G683, U692/G693, A702/G703; U788/U789; G925-C930) or the S7 protein (U957/A958, A1257, G1260; A1360).

## 3. Discussion

Omadacycline is a promising aminomethylcycline with therapeutic potential against severe infectious diseases. An in-depth mechanistic analysis is warranted, because resistance to first and second generation tetracyclines is already widespread [8,10], and resistance to TGC is being observed [38,39,40,41,42]. Structurally, OMC is more similar to TGC than to TET, since the former two are both derived from minocycline and carry a modification, albeit a different one, at the C9 position of the tetracycline D-ring (Figure 1). The OMC MIC values against the *E. coli* test strains, however, are more similar to TET than to TGC. They correlate nicely with the binding affinities of the respective drugs to *E. coli* ribosomes, which are also similar for TET and OMC [4,13], but 10–20-fold higher for TGC [4,17]. According to the crystal structures showing TGC bound to either *Thermus thermophilus* 70S ribosomes or 30S subunits, the tighter binding of TGC at the primary site-1 is likely to result from additional interactions formed between the 9-*t*-butylglycylamido substituent and the rRNA (in particular with C1054) [4,5]. Identical interactions would not be likely with the substituent of OMC, and TET completely lacks the C9 extension, which would explain their lower affinities. For example, OMC lacks the peptide bond (thus having inherently higher conformational flexibility) in which the amide nitrogen in TGC is the basis of several potential interactions with C1054 [4,5]. However, the *t*-butylaminomethyl sidechain of OMC also bears an amine nitrogen that, similar to TGC, might be protonated (theoretical pKa is 12.4 in OMC and 10.7 in TGC) and participate in a hydrogen bond with C1054 [5].

Despite these differences in binding affinities, all three compounds react identically to the tetracycline-resistance mutations in the 16S rRNA (1058 G→C and 966 G→U; Figure 1C) which affect the functionally important primary tetracycline binding site [2,3,4,5]. Their MIC levels are increased 4- to 8-fold in the mutant strains (Table 1), reproducing the published data for TET and TGC, done using a different protocol [15]. Binding of the three antibiotics to the primary site must, therefore, be very similar, which is the case for TET and TGC in their crystal structures of the 70S ribosome and the 30S ribosomal subunit [4,5]. So far, no tetracycline derivative has been identified which mediates resistance against these mutations. This is not astonishing, since the contacts between TET or TGC and the primary site all involve interactions of the minimum tetracycline pharmacophore with the invariant sugar-phosphate backbone of the rRNA [7]. Fortunately, the level of resistance these mutations mediate is low and their presence impairs cell growth. In fact, we have been unable to generate a viable *E. coli* strain which carries both mutations in its 16S rRNA (G. Fleischer, C. Heidrich, C. Berens; unpublished observations). 

Binding of all three compounds to the primary site is further supported by chemical probing data. DMS modification at C1054 is enhanced in the presence of all three antibiotics, Fe^2+^-mediated cleavage is detected at U965 in h31, at G1053, C1054, and at C1195 and A1197 in h34. Cleavage at U965 is most likely due to Fe^2+^ bound in place of the second Mg^2+^ (Figure 3F, Mg^2+^-2), which was not distinguished in the initial crystal structures [2,3], but was subsequently observed in proximity to bases in h31 [4,5]. This Mg^2+^ interacts directly with the phosphate group connecting the bases A965 (in *T. thermophilus*) and G966. Complexation of a second metal ion to the tetracycline A ring has been described, with the C4 dimethylamino group playing an important role [43]. Cleavage and protection in h34 (G1053 and C1054) is most likely mediated by Fe^2+^ bound to positions C-11/C-12 of the antibiotics. An equivalent Mg^2+^ ion in the crystal structures of TET [2,4] and TGC [4,5] interacts with the phosphate group connecting C1054 with A1055 and both phosphate groups flanking U1196 and G1197. The protection alteration in Fenton cleavage in h34 could reflect both a shielding of G1053/C1054 by tetracycline and/or a tetracycline-induced localized distortion that decreases cleavage of G1053/C1054 and enhances cleavage of C1195/A1197 close to the position of a structurally important Mg^2+^ ion (Figure 1C) that is constitutively present at this position [4].

**Figure 4:**
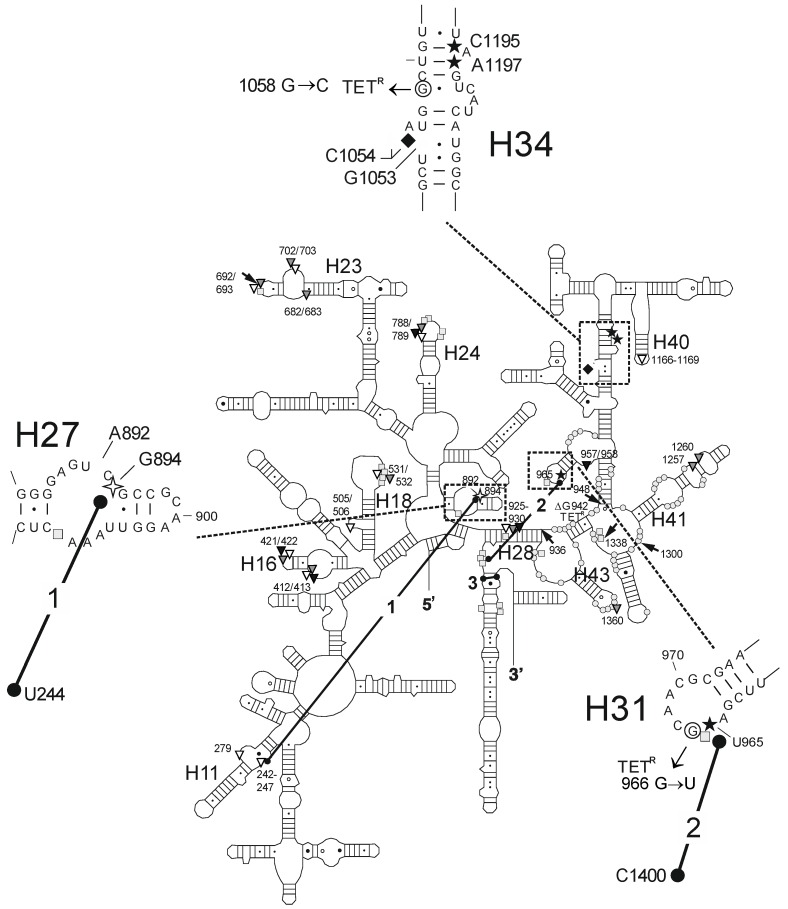
Summary of the interaction sites of TET, TGC, and OMC with the 16S rRNA. The secondary structure of the *E. coli* 16S rRNA is shown schematically [44]. Located within the stippled boxes and shown in more detail in the enlarged sections are bases that (i) display altered reactivity towards DMS probing in the presence of TET, TGC or OMC (white diamond: TET only; black diamond: all three) [15,25]; (ii) lead to weak resistance against TET, TGC, and OMC when mutated (TET^R^) [15,20,21,37]; show either (iii) Fe^2+^-mediated specific cleavage (white 4-pointed star, black star) [15], or (iv) protection from Fe^2+^-mediated cleavage in the presence of TET, TGC, and OMC (white 5-pointed star). In addition, the secondary structure contains (i) sites with altered reactivity towards DMS in the presence of tRNA (grey rectangle) [34] or the S7 protein (grey circle) [36]; (ii) direct photocrosslinks to TET (black arrow) [33,45]; (iii) RNA-RNA crosslinks affected by TET (black dumbbell) [35] or (iv) sites with Fe^2+^-mediated non-specific cleavage in the presence of TET (white triangle), TGC (grey triangle) or OMC (black triangle).

We also observed TET-dependent cleavage at G894 in helix h27. G894 is part of a secondary TET binding site (site-5: Figure 1B) [2,3], is close to A892, which is protected from methylation by DMS in the presence of TET [25], and is the site of a TET-inhibited crosslink to U244 [35]. This once again demonstrates excellent agreement between the Fenton cleavage data and published biochemical (crosslinks, chemical probing), genetic (resistance mutations) and structural data [2,3] (Figure 4). Fenton cleavage was not observed at this position in the previous study [15], but this might be due to the different experimental conditions used. Unlike TET, TGC and OMC show neither protection from DMS modification, nor Fenton cleavage at these positions. The initial enhancement seen at low concentrations of antibiotics might be due to indirect effects caused by drug binding to another site which affects exposure to DMS at A892. Since TGC is also not observed to bind to the secondary site [4,5], the simplest explanation would be that this site is not bound by TGC and OMC and, therefore, it represents a secondary tetracycline-binding site without biological activity.

Antibiotic-specific Fenton cleavage sites were not identified in h29 which is close to TET site-4 and site-6 (Figure 1B) [3]. Biochemical [33,45] and genetic [37] data suggests that tetracycline binds in this area and Fenton cleavage was detected in a previous study at nucleotides 1139–1341 [15]. Possibly, these cleavages are not observed here due to the respective experimental conditions in the two studies. Crystal structures of TET and TGC bound to 70S ribosomes [4] and of TGC bound to 30S ribosomal subunits [5] failed to find antibiotic at any of the secondary sites from the earlier 30S ribosomal subunit structures [2,3]. Photocrosslinking also yielded different results using either 70S ribosomes or 30S ribosome subunits [33,45], with crosslinks to 70S ribosomes occurring at TET concentrations of 40 µM and higher [33].

We did detect additional cleavage signals (Table 2, Figure S4, also summarized in Figure 4), but only at high concentrations of antibiotic. We attribute this to the different protocol used here, because such cleavage sites had not been observed before [15]. Most of these cleavage sites correlate well with sites of tetracycline binding found in earlier crystal structures [2,3], sites of altered reactivity towards DMS probing in the presence of TET, tRNA or the S7 protein [25,34,36], photo crosslinks involving either rRNA-rRNA [35] or TET-rRNA [33] and mutations leading to TET/TGC resistance [15,20,21] (Figure 4). Only the Fenton cleavages at positions A412/G413 and U421/C422, which were observed for all three antibiotics, and at positions G505/G506, which were observed for TET and TGC, do not correspond to any data from biochemistry, crystallography or genetic studies. The assumption that these signals are non-specific, due to their appearance at only the highest antibiotic concentration (125 µM), is supported by the observation that similarly high TET concentrations (i.e., 40–250 µM) were required to give distinct signals in the biochemical studies [25,33,35]. In further agreement with a non-specific nature of these cleavage sites, a mere four of the sixteen signals were obtained with all antibiotics. The remaining cleavage sites were detected for TET, TGC or both. The idiosyncratic nature of these cleavage events most likely reflects small differences in the structural and chemical properties of the three derivatives, due to their different modifications, allowing them to interact with different pockets in the 30S ribosomal subunit. In conclusion, OMC interacts with the ribosome like a typical tetracycline. It is susceptible to mutations in the 16S rRNA, like all other tetracycline derivatives tested so far. 

## 4. Materials and Methods

### 4.1. Materials

All chemicals were either from Sigma or Roth. Tetracycline-hydrochloride (#T7660) was from Sigma-Aldrich, tigecycline was from Pfizer (New York City, NY, USA), and omadacycline was supplied by Paratek Pharmaceuticals (Boston, MA, USA). Plasmid DNA was isolated using NucleoSpin Plasmid kits (Macherey & Nagel). DNA oligonucleotide primers were from Eurofins Genomics and [γ-^32^P]-ATP was from PerkinElmer.

### 4.2. Strains and Plasmids

*E. coli* DH5α (CGSC #12384; [46]) was routinely used as plasmid host. *E. coli* CAN/20-E12 (*rbn*, *rna*, *rnb*, *rnd*) [47,48] served as source for the 70S ribosomes. *E. coli* ATCC-25922 (ATCC #25922) was the quality control strain for the MIC determinations. *E. coli* TA527 (F^−^, *ara*, Δ*lac*, *thi*, Δ*rrnE*, Δ(*rrsB-gltT-rrlB*)101, Δ(*rrsH-ileV-alaV-rrlH*)103, Δ(*rrsG-gltW-rrlG*)30::*lacZ*^+^, Δ(*rrsA-ileT-alaT-rrlA*)34, Δ(*rrsD-ileU-alaU-rrlD*)25::cat^+^, Δ(*rrsC-gltU-rrlC*)15::*cat*^+^ *ilv*^+^) (CGSC #12282; [26]) was used to measure MIC values for TET, TGC, and OMC of mutated 16S rRNA in the absence of a wild type background. This strain is deleted for all seven operons encoding rDNA genes. For viability, *E. coli* TA527 therefore contains the pSC101 derivative pHK-rrnC^+^ bearing the entire *rrnC* operon from *E. coli*. The plasmid pKK3535, a pBR322 derivative which carries the 7.5-kb BamHI fragment from λrif^d^ containing the entire *rrnB* operon from *E. coli*, was taken as a wild type rRNA control [27]. The plasmid pKK1058C is a pKK3535 derivative bearing a point mutation of G→C at base 1058 of the 16S rRNA [20]. The plasmid pKK966U is a pKK3535 derivative bearing a point mutation of G→U at base 966 of the 16S rRNA [15]. The rRNA residues were numbered according to the *E. coli* scheme and helices indicated using the standard nomenclature throughout this manuscript [49]. Chemically-competent TA527/pHK-rrnC are transformed with either pKK3535 or one of its mutant derivatives. Transformants that grow on ampicillin (pKK3535) are checked for loss of kanamycin resistance indicating the loss of pHK-rrnC. This ensures a clean, homogenous genetic background for the rRNA to check the consequences of individual mutations in the rRNAs.

### 4.3. MIC Determinations

Antibiotic susceptibility testing was performed via the agar dilution MIC methodology, using Mueller Hinton Agar and following the recommendations of the Clinical and Laboratory Standards Institute (CLSI) as set out in documents M7-A8 and M100-S21, except that bacterial colony growth was evaluated after 40–44 h at 35 °C, due to the slower growth of the TA527 test strains. The longer incubation time did not affect the MIC values of the quality control strain ATCC-25922, which fall nicely into the permitted range of concentrations from Table 4A of the CLSI document M100-S21 (see Table 1). To prepare the inoculum, strains were grown to a 0.5 McFarland standard, which was determined by measuring the optical density at 625 nm. Ampicillin (100 µg/mL) for selection of the pKK3535-plasmids in the TA527 strain was added to the growth medium.

### 4.4. Isolation of 70S Ribosomes

Isolation of 70S ribosomes followed the protocol described previously [50] with modifications to scale the initial fermentation to 100 L, which yielded 89 g of *E. coli* CAN/20-E12. This cell pellet was suspended in 3 mL TICO buffer (10 mM HEPES-KOH, pH 7.6; 6 mM MgCl_2_; 30 mM NH_4_CI; 6 mM β-mercaptoethanol) supplemented with 0.25 mM phenylmethanesulfonyl fluoride per 1 g of cells. The cell suspension was French-pressed and the lysate cleared with two centrifugation steps; the first for 45 min at 30,000× g and the second for 17 h at 72,500× g. The pellet (crude ribosomes) from the second centrifugation step was resuspended in TICO buffer. A fraction containing 4000 A_260_ units of these crude ribosomes was loaded to a 5.7%–40% sucrose gradient (in TICO buffer), prepared in a 15 Ti Zonal rotor and centrifuged 17 h at 23,000 rpm. The gradient was fractioned and the fractions containing 70S particles were pooled, centrifuged at 85,000× g for 22 h and the washed pellet resuspended in TICO buffer at 600 A_260_/mL.

### 4.5. Chemical Modification of 70S Ribosomes with DMS

An amount of 25–30 pmol of 70S ribosomes in 48 μL TAKA_7_ buffer (50 mM Tris-HCl, pH 7.5; 70 mM NH_4_Cl; 30 mM KCl; 7 mM MgCl_2_) were incubated at 37 °C for 20 min, followed by incubation at ambient temperature for 5 min in the absence or presence of the respective antibiotic (final volume 49 μL). The dimethylsulfate (DMS) reaction was started by adding 1 μL DMS (1:10 dilution in 96% ethanol) and incubated at room temperature for 6 min. The reaction was stopped with 2 μL of β-mercaptoethanol (diluted 1:5 in water). The sample was precipitated by adding 2 μL glycogen (10 mg/mL) and 300 μL of a mixture of ethanol/0.3 M sodium acetate, pH 5.

### 4.6. Fenton-Mediated Hydroxyl Radical Cleavage Reactions

Fe^2+^-mediated hydroxyl radical cleavage reactions were carried out as described previously [51]. An amount of 4 μL of ribosomes (5 pmol per μL diluted in TAKA_7_), 1 μL of either a 10× antibiotic stock solution or H_2_O, and 2 μL of 5× NCB (125 mM MOPS-KOH, pH 7.0; 15 mM MgCl_2_; 0.5 mM spermidine) were incubated for 30 min at 37 ± 2 °C, followed by a 10 min incubation at room temperature. Then 1 μL of 2.5 mM FeCl_2_ was added to the reaction tube, mixed by centrifugation in a picofuge and incubated for 1 min before adding 1 μL of 12.5 mM sodium ascorbate. After 1 min incubation, 1 μL of 12.5 mM H_2_O_2_ was added and rapidly mixed to initiate the reaction. The final concentrations were 250 μM for Fe^2+^ and 1.25 mM for both sodium ascorbate and H_2_O_2_. Instead of FeCl_2_, 1 μL H_2_O were added to the control sample. In the Mg^2+^ competition experiments, MgCl_2_ was added as a 10× stock solution of the final Mg^2+^ concentration to the 1.25 mM FeCl_2_ solution. This mixture was then pipetted into the reaction tube and the cleavage reaction continued as above. The cleavage reaction was stopped after 1 min by adding thiourea to a final concentration of 125 mM. For precipitation, the sample volume was first increased to 100 μL with H_2_O. Then, 2 μL of a 10 mg/mL glycogen solution and 300 μL ethanol/0.3 M sodium acetate, pH 5, were added.

### 4.7. Extraction of rRNA

The rRNA was isolated as described [31]. The ethanol-precipitated pellets were resuspended in 200 μL RE-buffer (300 mM sodium-acetate; 0.5% (*w*/*v*) sodium dodecyl sulfate (SDS); 5 mM EDTA), supplemented with 8 Units RNAse Inhibitor (40 U/μL; Roche) and stored at 4 °C until use. Precipitated SDS was dissolved by gentle shaking at ambient temperature for 10 min. The ribosomal proteins were removed by successive phenol, phenol-chloroform-isoamylalcohol (25:24:1) and chloroform-isoamylalcohol (24:1) extractions. After a final centrifugation step at 15,000× g for 5 min, the RNA-containing solution (200 μL) was transferred to a new reaction tube and precipitated by adding 2 μL of glycogen (10 mg/mL) and 600 μL of a mixture of ethanol/0.3 M sodium acetate, pH 5. The rRNA was resuspended in 30 μL of DEPC-treated water and stored at −78 °C.

### 4.8. Primer Extension Reaction

The primer extension reaction was performed as described [31]. Briefly, 2 μL of the isolated rRNA (0.5–0.7 µg/μL) was mixed with 1 μL 5× Q-solution (Qiagen, One-step RT-PCR Kit) and 1 μL 5′-[^32^P]-labelled primer. The primers used were spaced approximately every 150 nucleotides on the 16S rRNA [52]. For better mapping of some of the cleavage sites, we used the following primers: 563rev (CGTGCGCTTTACGCCCAG), 704rev (CGGTATTCCTCCAGATCT), 938rev (ACCACATGCTCCACCGC), 1098rev (GGGTTGCGCTCGTTGCG), and 1256rev (TTGCTCTCGCGAGGTCGCT) instead of the primers #683, #906, #1199, and #1508 [52]. The hybridization was carried out by heating the mixture for 1 min at 94 °C followed by continuous cooling to 50 °C. The extension reaction was started by adding 10.5 μL extension-mix (2 μL 2.5 mM dNTPs; 3 μL 5× first strand buffer (Invitrogen); 1.5 μL 5× Q-solution; 1 μL 0.1 M DTT; 0.2 μL RNAse inhibitor (Promega); 0.2 μL SuperScriptIII reverse transcriptase (200 U/μL; Invitrogen); 2.6 μL DEPC-treated H_2_O) to 4.5 μL of the hybridization sample. The extension reaction was carried out in a final volume of 15 μL for 50–60 min at 50 °C. To stop the reaction, the RNA was degraded by adding 4 μL 1 M NaOH, followed by 30–60 min incubation at 42 °C. After neutralization by adding 4 μL of 1 M HCl, the cDNA was precipitated by the addition of 2 μL EDTA (0.5 M, pH 8), 2 μL glycogen (10 mg/mL), and 100 μL ethanol/0.3 M sodium acetate, pH 5. The samples were precipitated at −20 °C for 3–6 h or overnight. Following precipitation, the cDNA was washed with 80 μL 70% ethanol, the ethanol removed with a pipette and the pellet resuspended in 10 μL loading buffer (0.3% each of bromophenol-blue and xylene cyanol, 10 mM EDTA, pH 7.5, and 97.5% deionized formamide).

## Figures and Tables

**Table 1:** Minimal inhibitory concentrations of TET, TGC, and OMC for *E. coli* strains with tetracycline-sensitive and tetracycline-resistant ribosomes.

Test Compound	MIC Parameter	Value (µg/mL) against Each Bacterial Strain			
		ATCC-25922	TA527 (wt)	TA527/1058C	TA527/966U
Tetracycline	MIC (fold increase)	1	2	8 (4×)	16 (8×)
Tigecycline	MIC (fold increase)	0.06	0.25	1 (4×)	1 (4×)
Omadacycline	MIC (fold increase)	nd	2	8 (4×)	16 (8×)

**Table 2:** Fe^2+^-mediated and TET-, TGC- or OMC-directed cleavage sites on the 16S rRNA and the corresponding biochemical crystallography and genetic data.

Fe^2+^ Cleavage Sites ^a^	Specific/Non-Specific ^b^	TET-/TGC-Site ^c^	Biochemical and Genetic Data ^a^
G242-G247 (h11)	ns (T)	site-2/site-5	protection against methylation by DMS at A892 (TET) [25] and A909 (tRNA) [34]; TET-inhibited crosslink U244 × G894 [35]
A279 (h11)	ns (T)	site-2/site-5	protection against methylation by DMS at A892 (TET) [25] and A909 (tRNA) [34]; TET-inhibited crosslink U244 × G894 [35]
A412/G413 (h16)	ns (TGO)		
U421/C422 (h16)	ns (TGO)		
G505/G506 (h18)	ns (TG)		
U531/A532	ns (TG)		protection against methylation by DMS at G529-G532 (tRNA) [34]
G682/G683 (h23)	ns (T)		protection against methylation by DMS at G693 (tRNA) [34]
U692/G693 (h23)	ns (TG)		protection against methylation by DMS at G693 (tRNA) [34]
A702/G703 (h23)	ns (TG)		protection against methylation by DMS at G693 (tRNA) [34]
U788/U789 (h24)	ns (TGO)		protection against methylation by DMS at A790/G791/A794/C795 (tRNA) [34]
G894 (h27)	s (T)	site-2/site-5	protection against methylation by DMS at A892 (TET) [25] and A909 (tRNA) [34]; TET-inhibited crosslink U244 × G894 [35]
G925-C930 (h28)	ns (TGO)		protection against methylation by DMS at G928 (tRNA) [34]
U957/A958 (h30/h31)	ns (O)		enhanced methylation by DMS at G954 and A977-C980, protection against methylation by DMS at A983 (S7) [36]
U965 (h31)	s (TGO)	site-1	inhibition of crosslink A967 × C1400 [35]; A965U/G966U/A967C mutation: TET resistance in *H. pylori* [37]
G1053/C1054 (h34)	s (TGO)	site-1	enhanced methylation by DMS at C1054 (TET, TGC) [15,25]; G1058C mutation: TET resistance in *P. acnes* [20] and *B. hyodysenteriae* [22]
G1166-A1169 (h40)	ns (T)	site-3	
C1195/A1197 (h34)	s (TGO)	site-1	enhanced methylation by DMS at C1054 (TET, TGC) [15,25]; G1058C mutation: TET resistance in *P. acnes* [20] and *B. hyodysenteriae* [22]
A1257 (h41)	ns (G)		protection against methylation by DMS at A1256 (S7) [36]
G1260 (h41)	ns (G)		enhanced methylation by DMS at A1261 (S7 + S9) [36]
A1360 (h43)	ns (G)		protection against methylation by DMS at A1360 (S7) [36]

**^a^**: For position of bases in 16S rRNA, see Figure 4; **^b^**: s: specific; ns: non-specific; T: tetracycline; G: tigecycline; O: omadacycline; **^c^**: from *Thermus thermophilus* according to [2,3,4].

## Supplementary Materials

The following are available online at www.mdpi.com/2079-6382/5/4/32/s1, Figure S1: Fe^2+^-complexed with TET, TGC or OMC affects cleavage of bases in 16S rRNA, Figure S2: Mg^2+^ competes with Fe^2+^-mediated cleavage, Figure S3: Fe^2+^-complexed with TET, TGC or OMC cleaves bases in 16S rRNA non-specifically.

## Abbreviations

The following abbreviations are used in this manuscript:

TET	Tetracycline
TGC	Tigecycline
OMC	Omadacycline
aa	amino acid
tRNA	transfer RNA
rRNA	ribosomal RNA
MIC	minimal inhibitory concentration
CLSI	Clinical & Laboratory Standards Institute
DMS	Dimethyl sulfate
EDTA	Ethylenediaminetetraacetic acid
DEPC	Diethyl pyrocarbonate
cDNA	complementary DNA
