# Supplementary material for: The Novel Aminomethylcycline Omadacycline Has High Specificity for the Primary Tetracycline-Binding Site on the Bacterial Ribosome
Source: Antibiotics (Basel). 2016 Sep 22;5(4):32. doi: 10.3390/antibiotics5040032 (PMC5187513; doi:10.3390/antibiotics5040032)
Supplement: Supplementary file 1 [file antibiotics-05-00032-s001.pdf]

Supplementary Materials: The Novel Aminomethylcycline Omadacycline Has High Specificity for the Primary Tetracycline-Binding Site on the Bacterial Ribosome

Corina G. Heidrich, Sanya Mitova, Andreas Schedlbauer, Sean R. Connell, Paola Fucini, Judith N. Steenbergen and Christian Berens

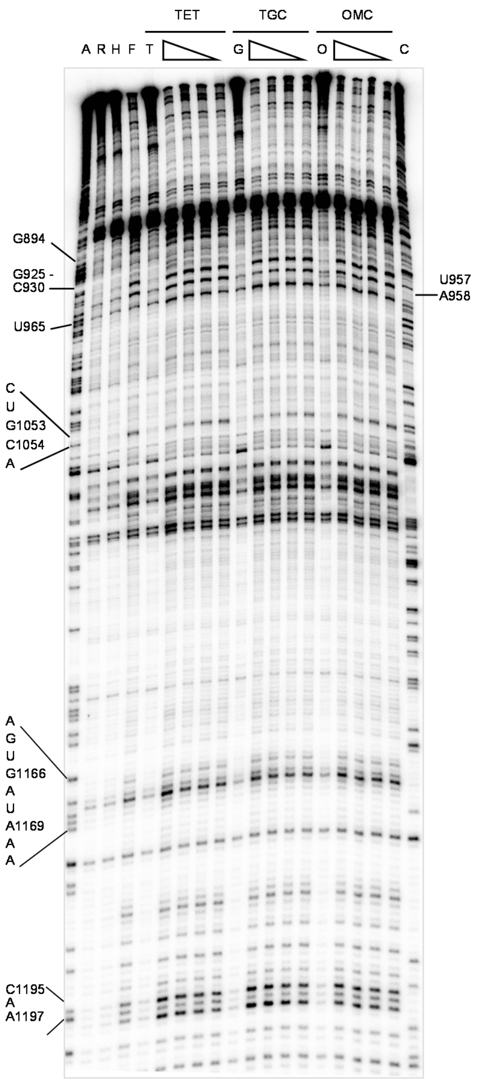

Figure S1. Fe^{2+}-complexed with TET, TGC or OMC affects cleavage of bases in 16S rRNA. Empty *E. coli* 70S ribosomes were incubated with increasing amounts of Fe^{2+}-complexed TET, TGC or OMC and incubated with sodium ascorbate and hydrogen peroxide. Sites of cleavage were detected by primer extension and analyzed by electrophoresis on denaturing 6% polyacrylamide gels with a typical gel being shown in the Supplemental Figure. Sites at which changes in cleavage intensity were found are highlighted. The dideoxy sequencing lanes are indicated with A and C; the unmodified RNA with R; Fe^{2+} incubated rRNA in the absence of sodium ascorbate and hydrogen peroxide with H; Fenton-cleaved rRNA in the absence of antibiotics with F; unmodified rRNA in the presence of 125 μM antibiotic; TET, TGC, OMC with T, G, and O, respectively; Fenton-cleaved rRNA in the presence of the respective antibiotic under the TET, TGC, and OMC headers where the wedge represents the presence of 125, 25, 5, and 1 μM of the respective antibiotic.

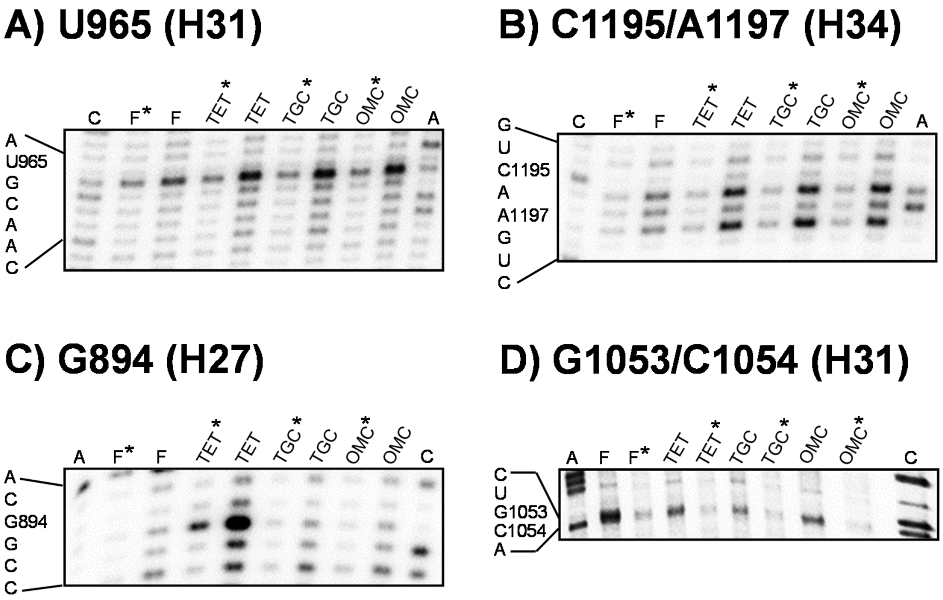

Figure S2. Mg^{2+} competes with Fe^{2+}-mediated cleavage. Empty *E. coli* 70S ribosomes were incubated with Fe^{2+}- or Mg^{2+}/Fe^{2+}-complexed TET, TGC or OMC and incubated with sodium ascorbate and hydrogen peroxide. Sites of cleavage were detected by primer extension and analyzed by electrophoresis on denaturing 6% polyacrylamide gels. Specific sites at which changes in cleavage intensity were found (Figure 3) are shown for (**A**) U965, (**B**) C1195/A1197, (**C**) A894, and (**D**) G1053/C1054. The dideoxy sequencing lanes are indicated with C and A; Fenton-cleaved rRNA in the absence of antibiotics with F; Fenton-cleaved rRNA in the presence of the respective antibiotic (25 μM) under the TET, TGC, and OMC headers. An asterisk (*) behind the symbol indicates that a 10-fold molar excess of Mg^{2+} was added as competitor to the Fe^{2+} solution.

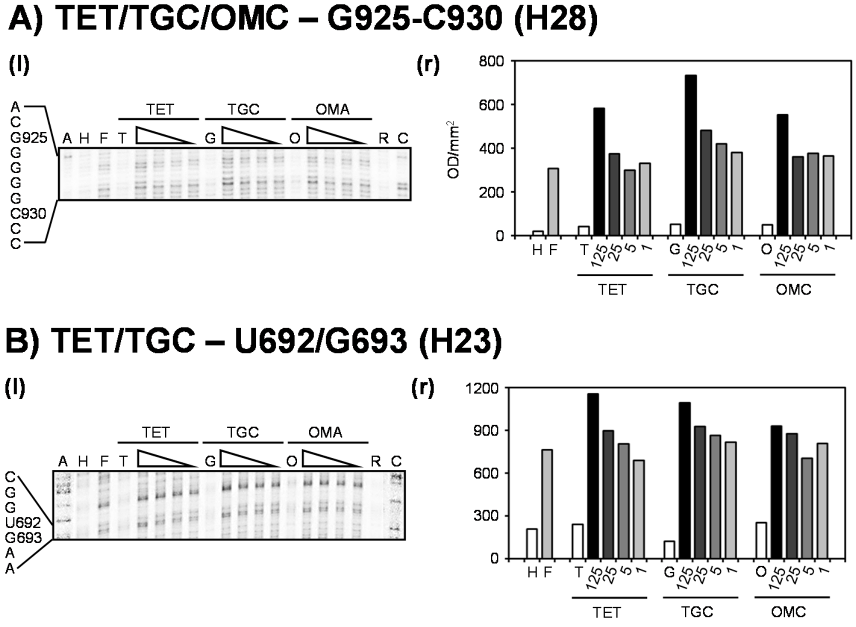

Figure S3. *Cont.*

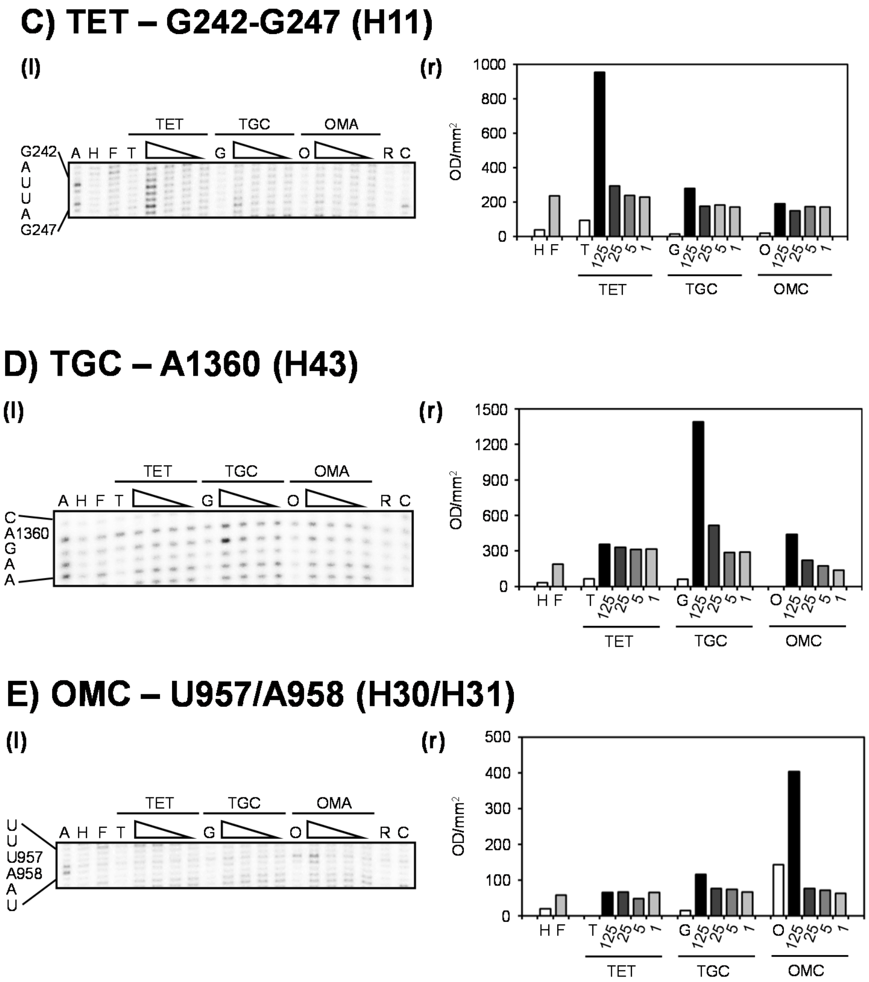

Figure S3. Fe^{2+}-complexed with TET, TGC or OMC cleaves bases in 16S rRNA non-specifically. Empty *E. coli* 70S ribosomes were incubated with increasing amounts of Fe^{2+}-complexed TET, TGC or OMC and incubated with sodium ascorbate and hydrogen peroxide. Sites of cleavage were detected by primer extension and analyzed by electrophoresis on denaturing 6% polyacrylamide gels, sections of which are shown to the left (l) of their respective quantification. Representative changes in cleavage intensity at the highest antibiotic concentration were found for TET, TGC, and OMC at nucleotides G925–C930 (**A**), for TET and TGC at nucleotides U692/G693 (**B**), for TET at nucleotides G242–G247 (**C**), for TGC at nucleotide A1360 (**D**), and for OMC at nucleotides U957/A958 (**E**). The dideoxy sequencing lanes are indicated with A and C; the unmodified RNA with R; Fe^{2+} incubated rRNA in the absence of sodium ascorbate and hydrogen peroxide with H; Fenton-cleaved rRNA in the absence of antibiotics with F; unmodified rRNA in the presence of 125 μM antibiotic; TET, TGC, OMC with T, G, and O, respectively; Fenton-cleaved rRNA in the presence of the respective antibiotic under the TET, TGC, and OMC headers where the wedge represents the presence of 125, 25, 5, and 1 μM of the respective antibiotic. The extent of rRNA cleavage in the presence of increasing amounts of antibiotic was quantified in a phosphorimager and is shown to the right (r) of the gel sections with a comparison to the control Fenton-cleaved rRNA in the absence of antibiotic (shown in lanes designated "F"). Quantification was adjusted for loading differences by normalization to regions unaffected by TET, TGC or OMC.

© 2016 by the authors; licensee MDPI, Basel, Switzerland. This article is an open access article distributed under the terms and conditions of the Creative Commons by Attribution (CC-BY) license (http://creativecommons.org/licenses/by/4.0/).
